# Prognostic and therapeutic insights into MIF, DDT, and CD74 in melanoma

**DOI:** 10.18632/oncotarget.28615

**Published:** 2024-07-19

**Authors:** Caroline Naomi Valdez, Gabriela Athziri Sánchez-Zuno, Lais Osmani, Wael Ibrahim, Anjela Galan, Antonietta Bacchiocchi, Ruth Halaban, Rajan P. Kulkarni, Insoo Kang, Richard Bucala, Thuy Tran

**Affiliations:** ^1^School of Medicine, Yale University, New Haven, CT 06520, USA; ^2^Department of Medicine, Section of Rheumatology, Allergy and Immunology, Yale University, New Haven, CT 06520, USA; ^3^Department of Dermatology, Yale University, New Haven, CT 06520, USA; ^4^Department of Dermatology, Oregon Health and Science University, Portland, OR 97239, USA; ^5^Cancer Early Detection Advanced Research Center (CEDAR), Portland, OR 97239, USA; ^6^Knight Cancer Institute, Oregon Health and Science University, Portland, OR 97239, USA; ^7^Department of Veterans Affairs Portland Health Care System, Operative Care Division, U.S. Portland, OR 97239, USA; ^8^Department of Medicine, Section of Medical Oncology, Yale University, New Haven, CT 06520, USA; ^9^Yale Cancer Center, Yale University, New Haven, CT 06520, USA

**Keywords:** MIF, DDT, melanoma, immune checkpoint inhibition, cancer transcriptomics

## Abstract

Macrophage Migration Inhibitory Factor (MIF) and its homolog D-dopachrome Tautomerase (DDT) have been implicated as drivers of tumor progression across a variety of cancers. Recent evidence suggests MIF as a therapeutic target in immune checkpoint inhibition (ICI) resistant melanomas, however clinical evidence of MIF and particularly of DDT remain limited. This retrospective study analyzed 97 patients treated at Yale for melanoma between 2002–2020. Bulk-RNA sequencing of patient tumor samples from the Skin Cancer SPORE Biorepository was used to evaluate for differential gene expression of MIF, DDT, CD74, and selected inflammatory markers, and gene expression was correlated with patient survival outcomes. Our findings revealed a strong correlation between MIF and DDT levels, with no statistically significant difference across common melanoma mutations and subtypes. Improved survival was associated with lower MIF and DDT levels and higher CD74:MIF and CD74:DDT levels. High CD74:DDT and CD74:MIF levels were also associated with enrichment of infiltrating inflammatory cell markers. These data suggest DDT as a novel target in immune therapy. Dual MIF and DDT blockade may provide synergistic responses in patients with melanoma, irrespective of common mutations, and may overcome ICI resistance. These markers may also provide prognostic value for further biomarker development.

## INTRODUCTION

Melanoma is one of the most aggressive and lethal forms of cancer, with an estimated 99,700 new cases in 2024 [1]. The development of immune checkpoint inhibitors (ICIs) has markedly transformed the landscape of cancer management and has since been established as the mainstay for several cancers, including advanced melanoma [2]. Anti-CTLA-4 inhibitors, which target regulatory T cells, and anti-PD-1/L-1 inhibitors, which target activated T-cells, dendritic cells, and tumor cells, have reshaped the management of melanoma leading to improvements in progression free and overall survival, with reports of upwards to 22% of patients experiencing complete response (CR) [3–9].

Amidst this dynamic landscape, the significance of macrophage migration inhibitory factor (MIF) and its structural homolog D-dopachrome tautomerase (DDT) have recently emerged as potential targets in immunotherapy. First identified in the 1960s as a macrophage migration inhibitor, MIF is an upstream immunoregulatory cytokine that contributes to tumorigenesis through its interactions with antigen-presenting cells and cytotoxic T lymphocytes in the tumor microenvironment (TME), and has been studied as a mediator of autoimmune diseases, such as rheumatoid arthritis [10–14]. Its versatile roles include the suppression of natural killer (NK) activity, promotion of tolerogenic dendritic cell activity, facilitation of T regulatory cell differentiation, and inhibition of T cell infiltration into tumors. MIF acts by activation of its cognate receptor CD74 and non-cognate receptors CXCR2, CXCR4, and CXCR7 [7, 9, 15, 16]. CD74, a non-polymorphic type II transmembrane glycoprotein present on the surface of immune and tumor cells, activates ERK, MAPK, and NF-κB signaling upon interaction with MIF and its coreceptor CD44 [17]. CD74 also functions independently of MIF, regulating MHC expression and antigen presentation, intracellular trafficking, and B cell selection and development [18]. DDT, also referred to as MIF-2, is less understood, but may have overlapping roles in promoting tumorigenesis and autoimmunity given its shared binding to CD74, CXCR4 and CXCR7 receptors [19]. MIF and DDT are ubiquitous among immune and non-immune cell types, though their effects on stromal and myeloid-derived cells, such as macrophages, dendritic cells and early myeloid derived suppressor cells (MDSCs), have been largely characterized in cancer progression [20]. MIF and DDT dysregulation have been implicated in most of the pathologic hallmarks of cancer, with downstream effects of proliferation, immune suppression, immune dysregulation, enhancement of angiogenesis and metastasis [21–23].

MIF and DDT have been studied extensively in a variety of preclinical and clinical cancer models, including those of hematologic, musculoskeletal, gastrointestinal, and gynecologic origin [24]. Among these, melanoma remains one of the most highly characterized. *In vitro*, elevated levels of MIF correlate with increased rates of growth and angiogenesis in human melanoma cell lines, and DDT knockdown correlates with reductions in proliferation markers and increases in apoptotic markers [25, 26]. *In vivo* murine studies suggest that MIF modulates immune evasion through upregulation of anti-inflammatory M2-type macrophages and lymphocytic infiltration of the TME, as well as by upregulation of Treg markers, T cell exhaustion, and reduction of lactate production, HIF-1α expression, and PDL-1 expression [27, 28]. In melanoma murine models, administration of the MIF small molecule inhibitor 4-IPP reduces tumor burden and metastasis, and the response is further augmented by co-administration of immune-checkpoint blockade antibodies [29]. Clinically, elevated serum levels of MIF have been observed in patients with advanced melanoma and correlate with poor response to anti-CTLA-4 therapy [28]. While murine studies have suggested that host-derived MIF drives tumor progression more than tumor-derived MIF, clinical data has correlated high stromal MIF expression with worse survival outcomes and can offer deeper mechanistic insight into its role at the site of tumor development [28, 30, 31].

Although DDT has been less studied than MIF in melanoma progression, studies investigating the efficacy of dual MIF and DDT inhibition in bladder cancer models have demonstrated greater antitumor effectiveness compared to either inhibitor alone, suggesting that targeting both MIF and DDT might yield superior clinical outcomes [32]. Taken together, MIF and DDT offer potential as therapeutic targets in patients with melanoma, including patients who progress on existing immune therapies. Furthermore, MIF and DDT hold potential as biomarkers to prognosticate response to ICI and survival in patients with melanoma.

Despite important therapeutic strides, melanoma is characterized by high mutational burden among solid tumors, and efforts to identify biomarkers with sensitivity and specificity for gauging immune checkpoint inhibitor response in melanoma patients remains an ongoing challenge. Currently, targeting the MIF/CD74 axis is a promising strategy for treating and overcoming resistance to immune checkpoint blockade therapy in melanoma and modulating the TME to promote effective antitumor immunity. However, most literature on the MIF/CD74 axis in melanoma is limited to cell and animal studies, with few studies reporting on clinical outcomes. Research evaluating DDT as a target in murine models remains sparse and, to our knowledge, no studies have evaluated DDT as a measurement of clinical outcomes of patients with melanoma [26]. Herein, we present the first study to retrospectively evaluate differential gene expression of MIF, DDT, and relevant pathway markers in regard to clinical outcomes in patients with melanoma.

## RESULTS

### Pre-processing of patient samples

**Figure 1:**
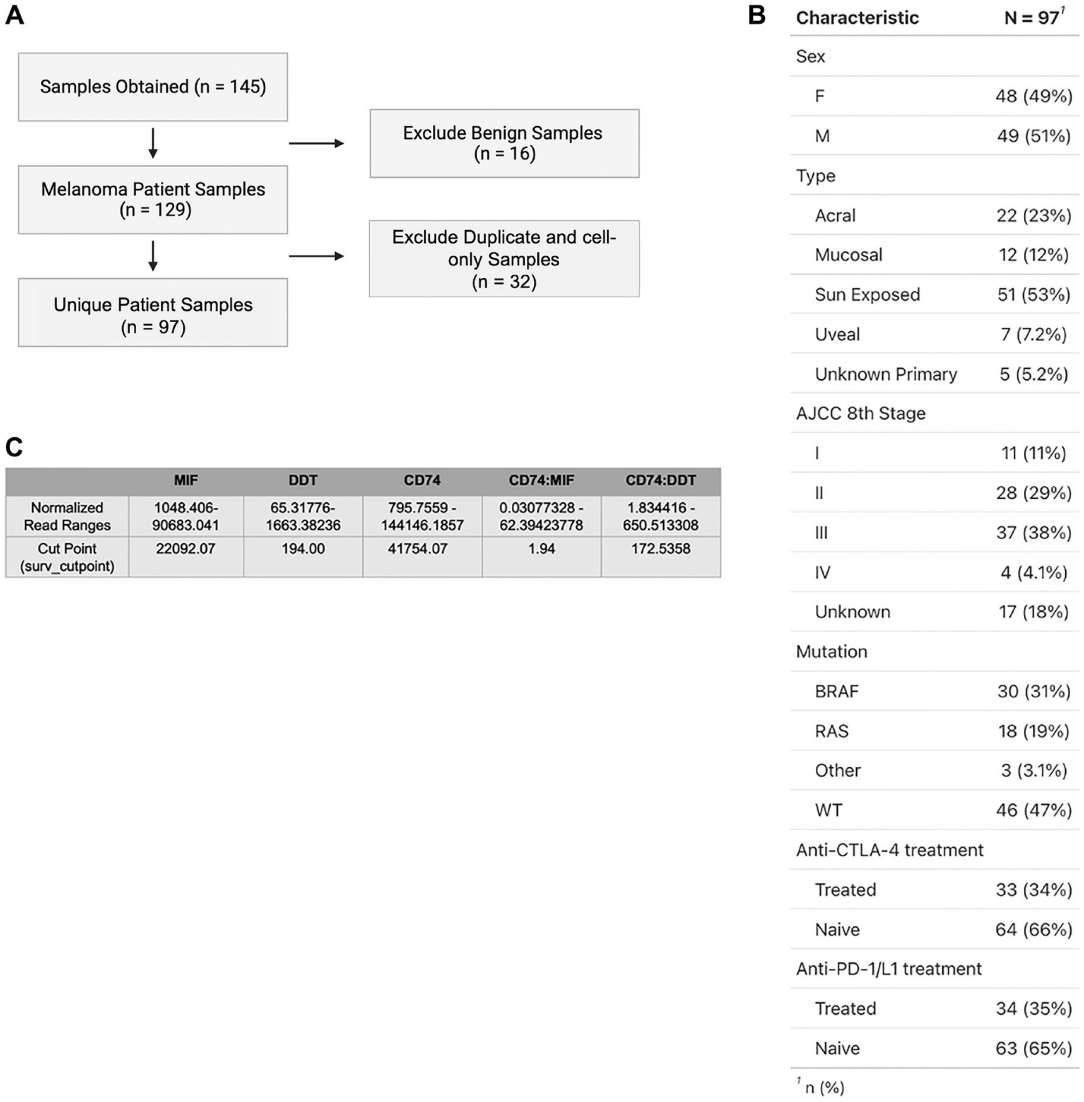
(**A**) Inclusion and exclusion criteria of samples from Yale Melanoma Biorepository used for analysis. A total of 145 tissue samples were obtained from the Yale Biorepository with corresponding bulk RNA sequencing. Benign skin samples (*n* = 16) were excluded prior to correlation analysis. Duplicate samples taken from the same patient and cell-only samples were excluded (*n* = 32) to generate a final subset of 97 unique patient samples used for subsequent analysis. (**B**) Demographic breakdown depicting age, subtype, mutational status, active treatment, and prior immunotherapy treatment status of unique patient samples. OTHER Mutations include C-KIT, GNAQ, and PRAME. (**C**) Table representation of normalized RNA read count ranges for MIF, DDT, CD74, CD74:MIF, and CD74:DDT, as well as corresponding cut points determined via surv_cutpoint function in survminer.

A total of 145 tissue samples were obtained from the Yale Biorepository with corresponding bulk RNA sequencing data. Control non-malignant skin samples (*n* = 16) were excluded prior to data analyses, and remaining tumor samples were used to generate correlation data. We excluded duplicate samples taken from the same patient and cell-only samples resulting from *in vitro* expansion of tumor (*n* = 32), to obtain findings that were reflective of the tumor and TME. The remaining unique samples (*n* = 97) included for analysis were then characterized by subtype (sun exposed, acral, mucosal, and uveal), mutation status (BRAF, RAS, C-KIT, GNAQ, PRAME, and wild type (WT)), and AJCC 8th Edition stage. Immune checkpoint inhibitors represented in our sample included anti-CTLA4 (ipilimumab) and anti-PD-1 (nivolumab, pembrolizumab) inhibitors. All inclusion and exclusion criteria as well as patient characteristics are outlined in Figure 1A, 1B.

### There is a direct correlation between MIF and DDT expression levels across melanoma mutations

**Figure 2:**
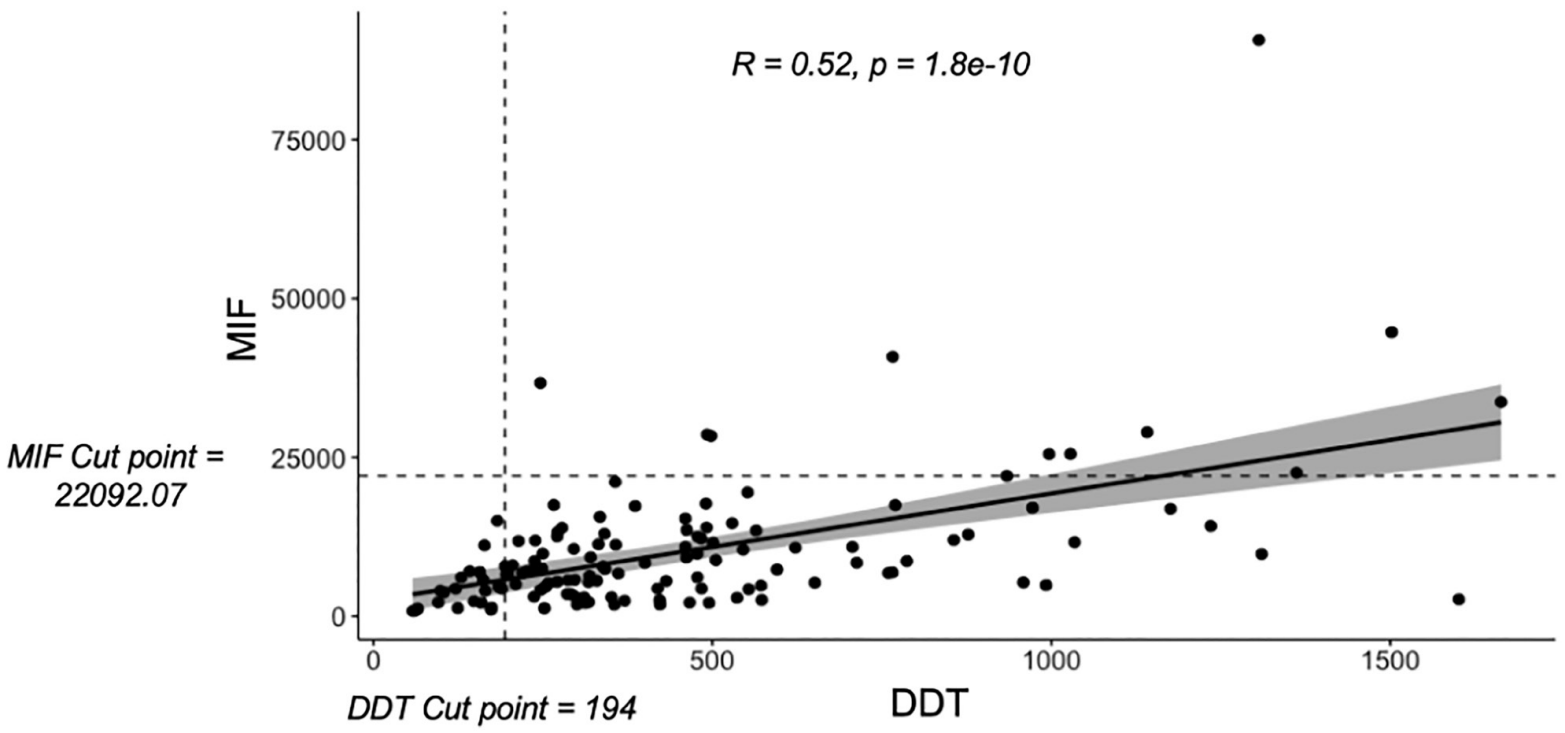
Linear correlation analysis revealed a direct correlation between MIF and DDT normalized read counts using spearman's rank test correlation test (*R* = 0.52, *p* = 1.8e-10, CI95: 0.3867525, 0.6394192). MIF and DDT cut points as determined by the surv_cutpoint function, subsequently used to generate high and low cohorts, are also visualized by the corresponding dashed lines.

Prior research has shown that MIF levels are elevated in patients with melanoma, but DDT levels have not been similarly explored. Accordingly, we first investigated the relationship between MIF and DDT levels in our cohort. Using Spearman’s rank correlation, we observed a direct correlation between MIF and DDT expression levels (R = 0.52, *p* = 1.8e-10, CI95: 0.3867525, 0.6394192) (Figure 2).

**Figure 3:**
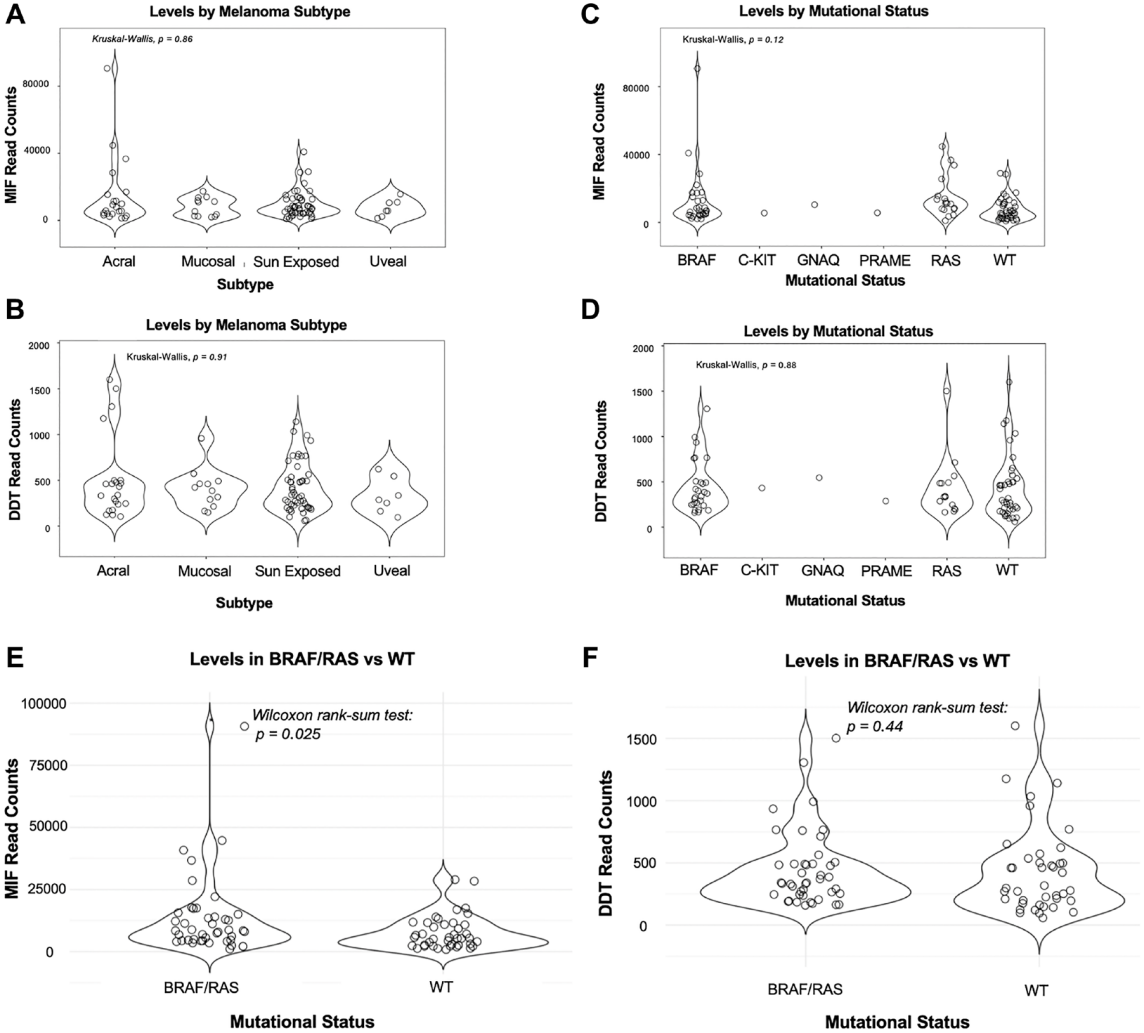
Kruskal-Wallis test applied to MIF and DDT levels across melanoma subtypes (acral, mucosal, sun-exposed, and uveal) and mutational status (BRAF, C-KIT, GNAQ, PRAME, RAS, and wild type (WT)). (**A**, **B**) There was no statistically significant difference in MIF or DDT normalized RNA read counts across common melanoma subtypes. MIF: *p* = 0.86; DDT: *p* = 0.91. (**C**, **D**). MIF and DDT expressions were elevated across mutational profiles, however there was no significant difference between groups. MIF: *p* = 0.12; DDT: *p* = 0.88. (**E**) MIF levels were elevated in combined BRAF/RAS groups when compared with WT groups on Wilcoxon rank sum analysis (p = 0.025). (**F**) There was no statistically significant difference in DDT levels between BRAF/RAS and WT groups (*p* = 0.44).

To assess differential gene expression across common melanoma subtypes (acral, mucosal, sun exposed, and uveal), we analyzed MIF and DDT levels using the Kruskal-Wallis test. We observed no statistically significant difference in MIF or DDT read counts across the subtypes (MIF *p* = 0.86, DDT *p* = 0.91) (Figure 3A, 3B). Pairwise Wilcoxon rank tests for MIF and DDT revealed no statistically significant differences between subtypes (Data not shown). Furthermore, investigation of MIF and DDT expression levels across common melanoma mutational profiles using the Kruskal-Wallis test revealed enrichment across all groups, though findings were not statistically significant (MIF *p* = 0.12, DDT *p* = 0.88) (Figure 3C, 3D). Upon combining BRAF and RAS groups, we observed significantly higher levels of MIF in the combined BRAF/RAS group when compared with WT groups on Wilcoxon rank sum analysis (*p* = 0.025) (Figure 3E). Conversely, there was no statistically significant difference in DDT levels between BRAF/RAS and WT groups (*p* = 0.44) (Figure 3F).

### Lower DDT and MIF expression levels correlate with better overall survival

**Figure 4:**
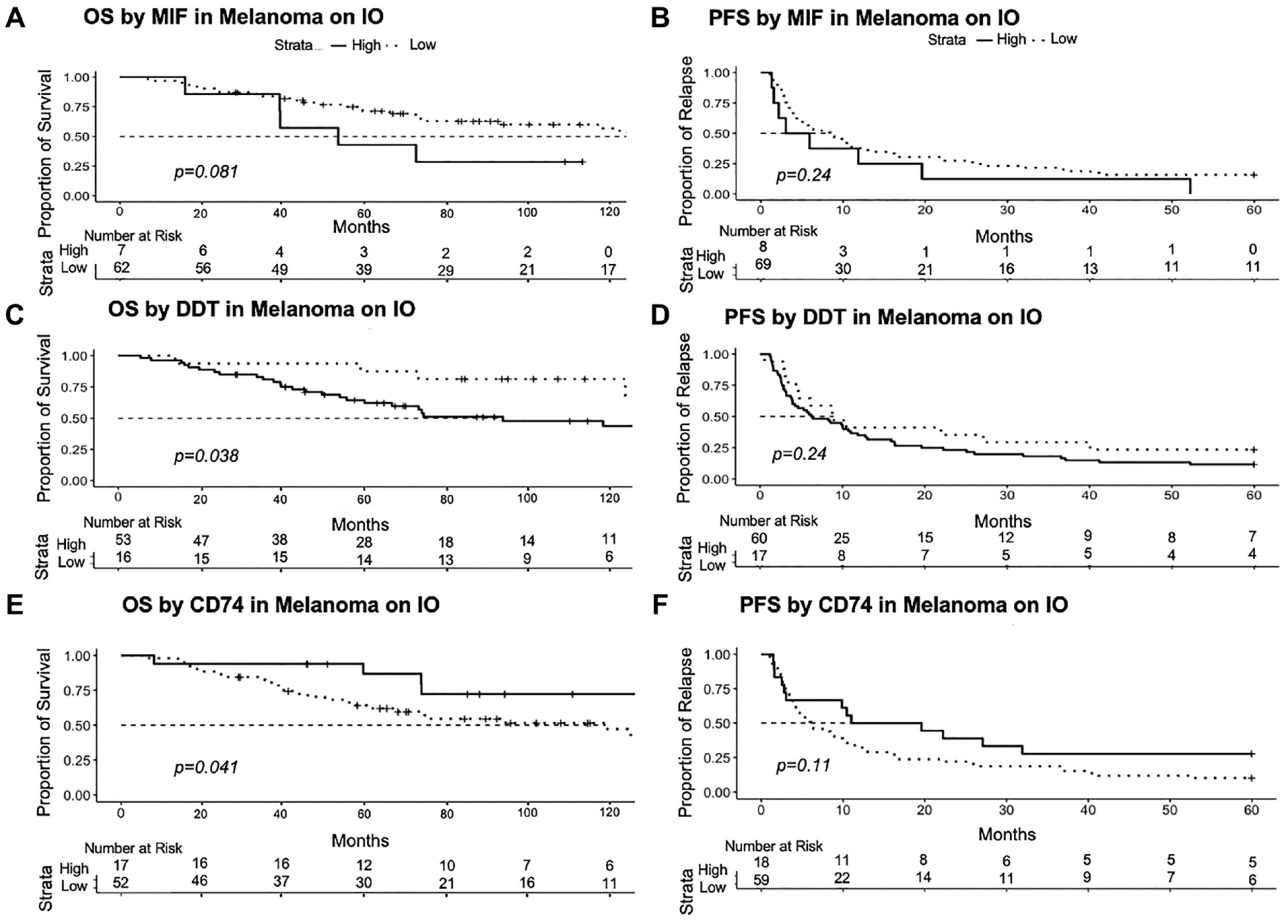
PFS and OS analyzed according to MIF, DDT, and CD74 using the surv_cutpoint approach in Rstudio. (**A**, **B**) Reduced MIF levels are associated with improved PFS (*p* = 0.24) and OS (0.081). (**C**, **D**) Reduced DDT levels are similarly associated with improved PFS (*p* = 0.24) and OS (*p* = 0.038). (**E**, **F**) Conversely, elevated CD74 levels are associated with improved PFS (*p* = 0.11) and OS (*p* = 0.041). Number at risk refers to the quantity of eligible patients alive at each time point. Abbreviation: IO: Immunotherapy.

We then investigated how MIF and DDT expression levels correlated with patient progression free survival (PFS) to their active treatment at time of sample collection and overall survival (OS). To maintain a consistent patient distribution in high and low DDT/MIF cohorts across analyses, patients were stratified using survminer cut points based on the OS data. This approach produced cut points of 22092 for MIF, 194 for DDT, 41754 for CD74, 1.94 for CD74:MIF, and 172.53 for CD74:DDT (Figure 1C). The expression cut point was then used to differentiate low and high levels for graphing PFS. Sample sizes in each group varied slightly, as data was censored if missing. We found that lower levels of MIF were associated with non-significant trends towards improved PFS (*p* = 0.24, *n* = 8 high MIF vs. *n* = 69 low MIF) and OS (*p* = 0.081, *n* = 7 high MIF vs. *n* = 62 low MIF) (Figure 4A, 4B). Interestingly, when determining cut points using the surv_cutpoint approach on the PFS data, a larger number of patients were grouped into high MIF (*n* = 66) when compared to low MIF (*n* = 11), and lower expression levels of MIF were associated with significantly improved PFS (*p* = 0.047) (Supplementary Figure 1). We similarly identified the number of patients at risk in the high and low DDT cohorts for PFS (*n* = 60, *n* = 17, respectively) and OS (*n* = 53, *n* = 16, respectively). When analyzing DDT levels, we similarly found that lower levels of DDT were associated with a trend towards improved PFS (0.24) and significantly improved OS (*p* = 0.038) (Figure 4C, 4D). Similarly, when applying the surv_cutpoint approach to PFS data, the trend for lower expression levels of DDT being associated with improved PFS was similar (*p* = 0.14) (Supplementary Figure 1).

### Higher CD74 expression levels are associated with improved progression-free and overall survival

We then investigated the relationship between CD74 expression levels and patient PFS and OS. Patients in the high CD74 cohort (*n* = 18) had a trend towards improved PFS (*p* = 0.11) when compared to patients in the low CD74 cohort (*n* = 59). Patients with higher levels of CD74 (*n* = 17) had significantly improved OS (*p* = 0.041) when compared with patients in the low CD74 cohort (*n* = 52) (Figure 4E, 4F). Application of surv_cutpoint to PFS data produced similar results, but did not reach statistical significance.

### Higher CD74:MIF and CD74:DDT expression ratios are associated with improved progression-free and overall survival

**Figure 5:**
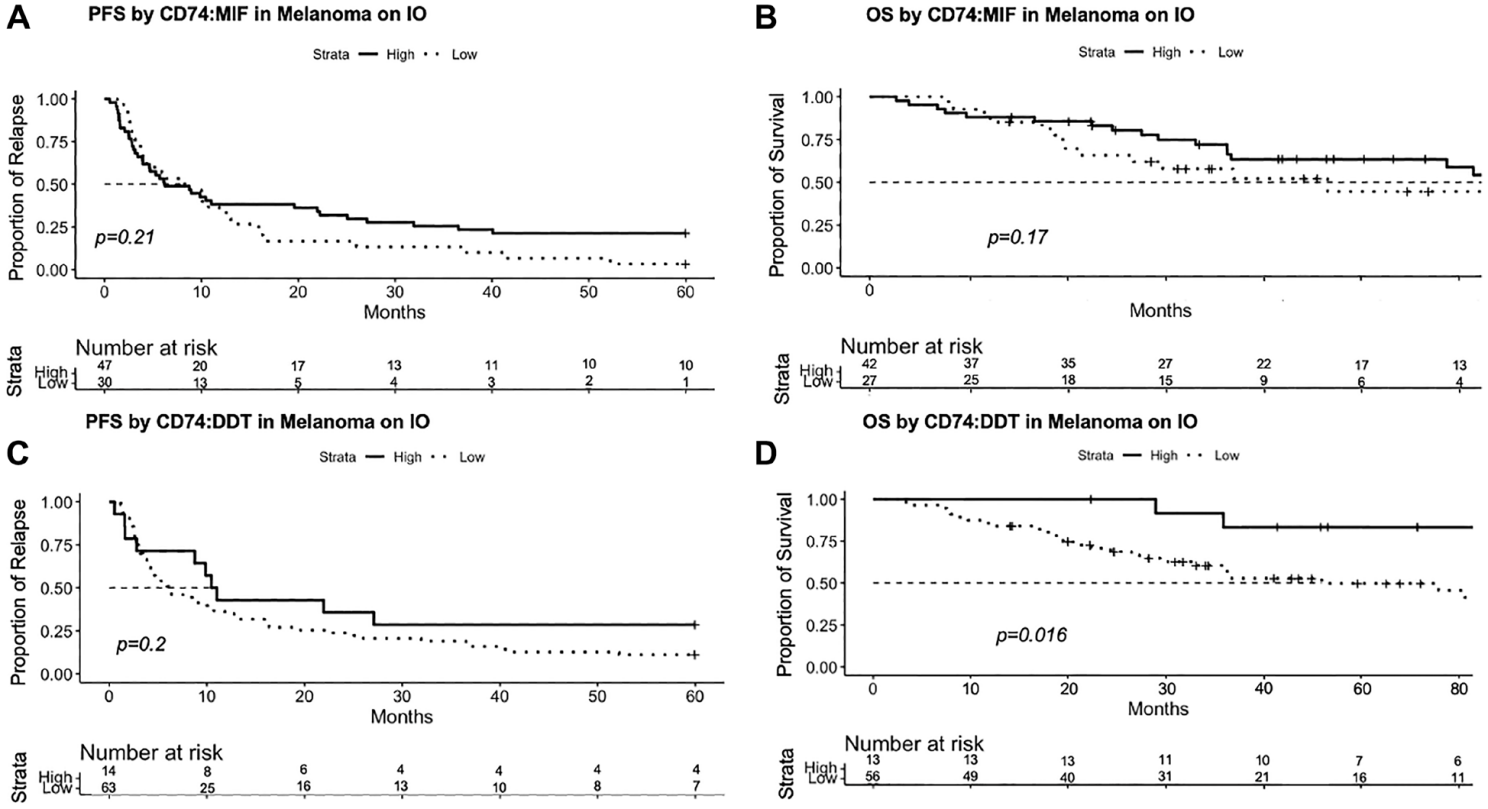
PFS and OS analyzed according to CD74:MIF and CD74:DDT expression ratio using the surv_cutpoint approach in Rstudio. (**A**, **B**) Elevated CD74:MIF levels are associated with improved PFS (*p* = 0.21) and OS (0.17) though results were not statistically significant. (**C**, **D**) Similarly, elevated CD74:DDT levels are associated with improved PFS (*p* = 0.2), as well as a significant improvement in OS (*p* = 0.016). Number at risk refers to the quantity of eligible patients alive at each time point. Abbreviation: IO: Immunotherapy.

As CD74 is found primarily on immune subsets where it is associated with improved outcomes in melanoma, we evaluated the ratio of CD74 to its ligands MIF and DDT. Past work has shown that the CD74:MIF ratio in the circulation can distinguish clinical subtypes of disease or disease progression in autoimmune hepatitis when compared with primary biliary cirrhosis, and work by Ekmekcioglu et al. has shown that CD74:MIF has the potential to prognosticate clinical outcomes in patients with melanoma [17, 33]. In our studies, the ratio of CD74 to either MIF or DDT was evaluated by dividing the normalized expression levels of each respective transcript. We found that patients in the high CD74:MIF cohort (*n* = 47) had a trend towards improved PFS when compared with those in the low CD74:MIF cohort (*n* = 30), although this was not statistically significant (*p* = 0.21) (Figure 5A). Patients in the high CD74:MIF cohort (*n* = 42) also were associated with a trend towards improved OS (*p* = 0.17) when compared with those in the low CD74:MIF cohort (*n* = 27), although this again did not reach statistical significance (Figure 5B). Patients in the high CD74:DDT (*n* = 14) cohort had improved PFS when compared with PFS in the low CD74:DDT cohort (*n* = 63), though not statistically significant (*p* = 0.2) (Figure 5C). We observed that samples in the high CD74:DDT cohort (*n* = 13) were associated with improved OS (*p* = 0.016) when compared with OS in the low CD74:DDT cohort (*n* = 56) (Figure 5D). These data suggest that the ratio of CD74:MIF and CD74:DDT expression in melanoma may provide prognostic value and potentially serve as clinical biomarkers for patients with melanoma.

### High CD74:MIF and CD74:DDT expression ratios are associated with increased expression of inflammatory and memory markers and enrichment in immune-related pathways

**Figure 6:**
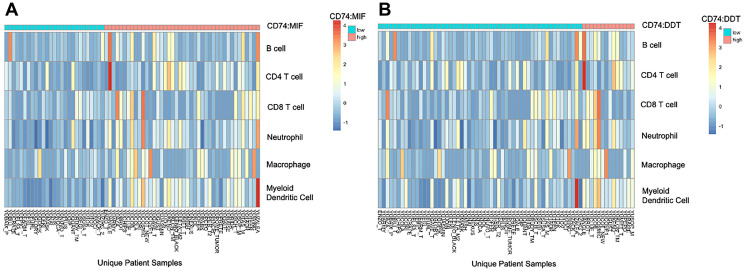
Tumor infiltrating immune cell profiling according to high (cyan) and low (salmon) CD74:MIF levels (**A**) and CD74:DDT levels (**B**) using TIMER2.0 deconvolution analysis. Cut points for high and low values was determined using the surv_cutpoint approach on Rstudio. An increase in intratumoral inflammatory markers are evident in high CD74:MIF and CD74:DDT cohorts when compared to their respective low cohorts.

Finally, we employed deconvolution analysis using TIMER to determine the relative immune cell type abundances in high and low CD74:DDT and CD74:MIF samples. We observed a relative over-representation of B cells, CD4+ and CD8+ T cells, neutrophils, myeloid dendritic cells, and macrophages in patients with high CD74:MIF and CD74:DDT levels compared to their low CD74:MIF and CD74:DDT counterparts (Figure 6). Enrichment in immune-related cell types was similarly observed across alternative algorithms (CIBERSORT, CIBERSORT abs.mode, EPIC, MCP-Counter, quanTIseq, TIMER, and XCell approaches) (Supplementary Figures 2 and 3).

**Figure 7:**
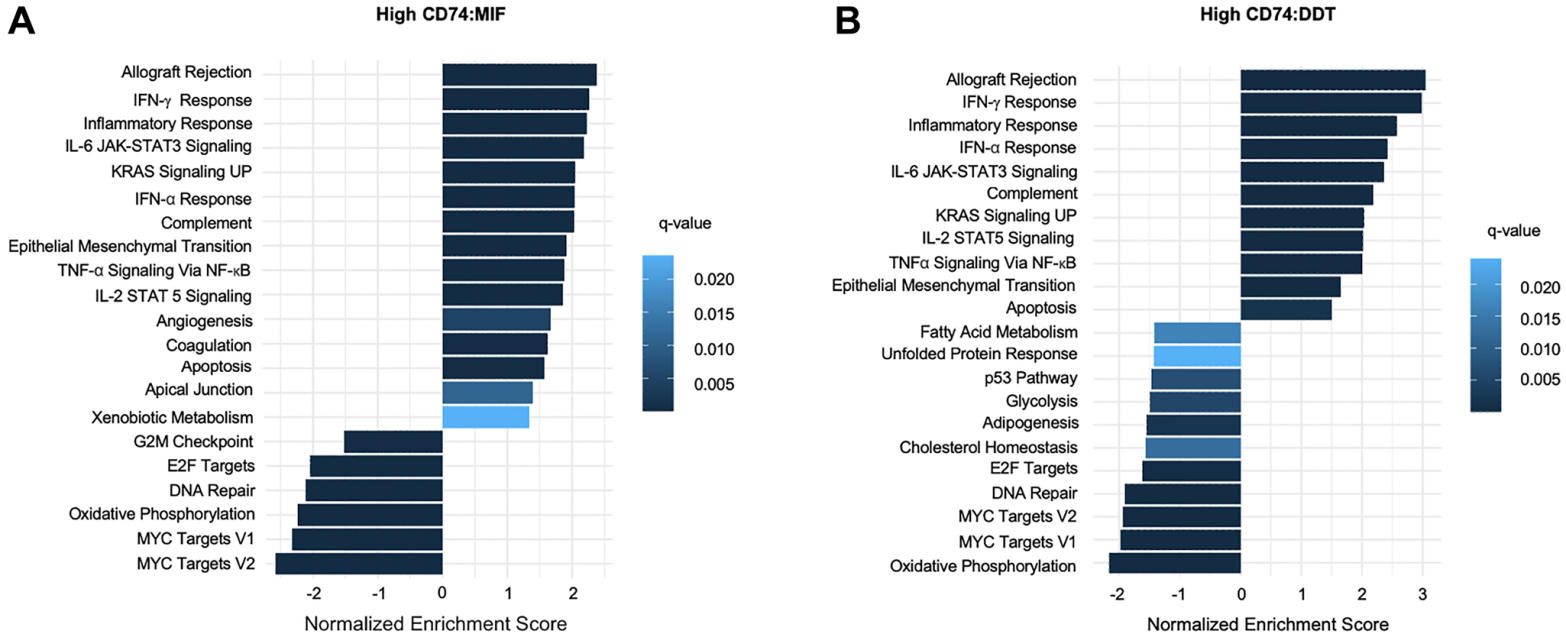
Gene Set Enrichment Analysis (GSEA) bar plot representation of (**A**) high CD74:MIF and (**B**) high CD74:DDT differentially expressed OS data using MSigDB Hallmarks gene collection set. Differential expression analysis reveals an upregulation of inflammatory-related pathways in reference to corresponding low CD74:MIF and low CD74:DDT data.

Gene set enrichment analysis (GSEA) of differentially expressed genes (DEGs) in the high CD74:MIF cohort similarly revealed an enrichment of pathways related to inflammation, allograft rejection, and interferon gamma response (Figure 7). KEGG Pathway analysis corroborated these results, highlighting enrichment of pathways involved in antigen presentation, NK cell activity, and T cell activation, such as MHC Class I and II, CD3/4/8/28, IFN-γ, and IL-2 (Supplementary Figures 4 and 5). Similar results were observed with GSEA of CD74:DDT DEGs. Moreover, a shared subset of high CD74:MIF and high CD74:DDT DEGs revealed a high concordance of similarity between upregulated and downregulated genes (Supplementary Figure 6). A complete list of shared DEGs which are significantly (*p*adj < 0.05) up-regulated (log2FC > 1.5) or down-regulated (log2FC < −1.5) are listed in Supplementary Table 1.

## DISCUSSION

Our study significantly expands on prior work by De Azevedo et al. by encompassing a larger cohort of individuals, coupled with a comprehensive approach to defining high and low MIF and DDT expression. Our study analyzed 97 unique samples from patients with melanoma who were treated at Yale between 2002–2020. Bulk RNA sequencing revealed patients had increased expression of both MIF and DDT with a direct correlation between the two. Furthermore, we found that decreased DDT and MIF levels were associated with improved OS and a trend towards improved PFS. Given the limited number of samples and treatment differences, we were not able to stratify the data based on the type of ICI at the time of sample collection. Analysis revealed elevated CD74 levels were associated with improved OS and PFS; and when we analyzed patient outcomes according to CD74:MIF and CD74:DDT expression ratios, we found that higher values were associated with improved OS and PFS. Finally, deconvolution analysis revealed that high CD74:MIF and CD74:DDT ratios were associated with increased expression of known pro-inflammatory markers and enrichment of immune cells suggestive of greater immune cell infiltration.

Prior studies have shown that MIF expression levels are elevated in patients with melanoma, however no prior studies have reported simultaneous DDT expression levels in these patients [25, 28, 34]. Our findings of elevated MIF and DDT levels were consistent with our hypothesis, based on prior arguments that DDT may have similar pro-inflammatory and pro-tumorigenic properties given its homology to MIF, shared signaling through their shared cognate receptor CD74, and known similar downstream signaling via the ERK1/2 MAP kinase cascade. Additionally, this data is supported by prior animal studies suggesting a therapeutic effect of MIF or DDT antagonism in mouse models of melanoma [26].

Findings from our survival analysis are consistent with existing literature demonstrating that increased MIF levels are associated with worse prognosis in patients with melanoma; particularly in patients with advanced disease or evidence of metastases. To our knowledge, the present study is the first to report a similar finding for DDT. Our study observed a direct correlation between CD74 expression levels and improved OS and PFS, reflecting its role in enacting immune cell development, selection, maturation, and antigen presentation. These data are supported by recent work in glioblastoma which has shown that inhibition of the MIF/CD74 axis with anti-CD74 inhibitor Ibudilast reduces the monocytic subset of MDSCs and enhances intratumoral CD8 T cell activity [35]. Similarly, our results suggest that high CD74 expression in the context of low MIF expression promote pro-inflammatory responses such as TNF-α signaling and apoptosis within the TME. These data are consistent with prior studies reporting that soluble CD74 within the TME improves survival in patients with malignancy, particularly melanoma, via MIF-CD74 inhibition and enhancement of pro-apoptotic pathways [34]. Our findings are in line with those of Ekmekcioglu et al., who similarly observed that high CD74, low MIF, and high CD74:MIF levels correspond to improved OS. However, unlike their study, we did not observe improvement in PFS in our high CD74:MIF group. In contrast, data on CD74:DDT has not yet been reported, though our results suggest that the CD74:DDT expression ratio offers similar prognostic value. Taken together, our data suggests measurement of CD74:MIF and CD74:DDT expression level ratios can provide prognostic information with respect to survival in patients with autoinflammatory disease and melanoma better than MIF, DDT, or CD74 alone [34].

It should be noted that while our PFS data for MIF, DDT, CD74, CD74:MIF, and CD74:DDT did not reach statistical significance, the overall data trends may be expected and warrant further study with additional patient cohorts or melanoma subtypes. Interestingly, application of PFS-derived surv_cutpoint functions to these data generated reverse results, in which high MIF and DDT cohorts exhibited a worse PFS compared to their low MIF and DDT counterparts. It should be noted, however, that the cut point values generated through these approaches differed markedly from those generated from OS data. From real-world experience, PFS does not necessarily translate to OS, which remains the gold standard to understand if interventions produce long-term outcomes and improve mortality. We therefore opted to prioritize OS data to define cut points, as it more meaningfully informs how the RNA data may be used as prognostic biomarkers.

Profiling of intratumoral MIF and DDT gene expression via deconvolution analysis further revealed enrichment of immune cells in patients with higher CD74:MIF and CD74:DDT level ratios when compared to their low CD74:MIF and CD74:DDT counterparts. It is known that MIF and DDT influence the TME to enhance tumorigenesis by modulating environmental signals from a predominantly pro-inflammatory to tumor-permissive phenotype. Our data corroborates prior reports of MIF and DDT triggering cytokines related to antigen presentation, T cell differentiation and NK cell activity, and tumor-associated macrophage infiltration, particularly expression of M2-type immunosuppressive macrophages [36–39].

Pharmacologic blockade of the MIF/DDT-CD74 signaling axis could potentially alter the immunosuppressive TME and offer new therapeutic options for melanoma. Targeting MIF and DDT may further offer benefit to patients with melanoma subtypes that are traditionally unresponsive to standard therapy, such as uveal, mucosal, and acral subtypes, and tumors harboring different mutational signatures [40]. The intraocular environment for instance, is an immunoprivileged site with high levels of TGF-β that contribute to suppression of NK cell activity and is therefore conducive to the survival of melanoma cells [41–43]. While little research has described the role of MIF in acral melanoma, it has similarly been hypothesized to drive pathogenesis via the presence of M2-type macrophages in the TME [44]. Given our observations that MIF and DDT gene expression levels do not correlate with melanoma subtype or mutational status and that high CD74:MIF and CD74:DDT expression level ratios correspond with enhanced intratumoral immune cell infiltration, clinical targeting of MIF and DDT holds potential to be effective across subtypes and mutational status.

Our study is the first to report survival findings in association with intratumor DDT expression and CD74:DDT expression level ratio. Thus, CD74:MIF and CD74:DDT expression ratio measurements offer promise as prognostic markers for survival outcomes and ICI response in patients with melanoma. Additionally, as our PFS analyses selected for patients who were on ICI at the time of biopsy, CD74:MIF and CD74:DDT also may have the potential to prognosticate patient response to ICI much better than MIF or DDT alone.

The retrospective nature of this study limited data collection to information provided in patient charts, and we lacked the ability to identify patients who were lost to follow up, transitioned care to an outside hospital, or patients who were unknowingly deceased. While samples that were included for PFS analyses were either on active or prior immune-therapy, there was high variability across patients regarding type of immune-therapy, length of treatment exposure, and time between treatment initiation and biopsy collection, limiting our ability to analyze response to treatment and PFS in these cohorts. Furthermore, we cannot account for potential downstream changes in MIF, DDT, or CD74 RNA levels secondary to active therapy. Additionally, our study assumes that patients do not have significant existing autoimmune comorbidities, for which MIF and DDT are known to play prominent roles in progression [45–48]. Patients with pre-existing autoimmune diseases have widely been excluded from prospective clinical trials due to the risk of development of immune-related adverse events, leaving little evidence to support the use of ICI therapy in this patient population [49–51]. Finally, bulk RNA sequencing provides us limited information regarding MIF, DDT, and CD74 expression, function, and interactions at the protein level. However, these data suggest that bulk RNA sequencing performed on biopsied tumors nevertheless may provide valuable prognostic information regarding survival outcomes, particularly overall survival.

Amidst growing evidence to suggest important roles for MIF and DDT in tumorigenesis, DDT remains far less understood. Further *in vitro* and *in vivo* studies are needed to validate the role of DDT in melanoma progression. Continued investigation of the molecular interactions between DDT and canonical and non-canonical receptors and effects on downstream signaling may elucidate features that are unique from MIF and provide further insight into the added benefit of a dual-MIF/DDT inhibition approach in cancer treatment using murine models. Genetic, transcriptomic, and proteomic analyses of DDT, CD74:MIF, and CD74:DDT in patients with melanoma can further shed light on the differential expression among varying disease stages, tumor subtypes, mutational statuses, and ICI use.

Our data bolsters existing evidence on the intratumoral effects of MIF and DDT on tumor permissiveness, primarily through immune modulation, with evidence translating to melanoma prognosis. In light of growing evidence that MIF blockade can overcome resistance to ICI therapy in patients with melanoma, our data further suggests that MIF and DDT exhibit potential as therapeutic targets and biomarkers in predicting response to treatment and survival, with CD74:MIF and CD74:DDT offering promise as markers of ICI response in those undergoing treatment. Further investigation is needed to understand the role and functions of DDT in the melanoma microenvironment in addition to its disparate, non-overlapping functions in tumorigenesis.

## MATERIALS AND METHODS

### Retrospective chart review

We analyzed bulk RNA sequencing previously obtained from 152 biopsies of patients treated at Yale for melanoma between 2002 and 2020 in collaboration with the Specialized Program of Research Excellence (SPORE) in Skin Cancer (Protocol #0609001869). Biopsies were obtained from patients undergoing surgical resection, irrespective of treatment history. All patients provided written consent. Pathology was reviewed to verify Breslow tumor depth, ulceration status, number of lymph nodes involved, presence of in-transit or satellite metastases, presence of distant metastases, and melanoma subtype (cutaneous, acral, mucosal, and uveal). Criteria from the 8th edition AJCC were then applied to unify and update staging. Time from ICI initiation to first immune-related toxicity, disease recurrence, and brain metastases were collected. Patient progression-free survival (PFS), response to therapy by tumor imaging metrics core (TIMC), and overall survival (OS) were also collected.

### Bulk RNA sequencing

Patient biopsies collected from the Skin Cancer SPORE Biorepository were evaluated by bulk RNA sequencing. For our patient cohort, rRNA was depleted starting from 25–1000 ng of total RNA using the Kapa RNA HyperPrep Kit with RiboErase (KR1351). Indexed libraries that met appropriate cut-offs for both quantity and quality were quantified by qRT-PCR using a commercially available kit (KAPA Biosystems) and insert size distribution was determined with the LabChip GX or Agilent Bioanalyzer. Samples with a yield of ≥0.5 ng/ul were used for sequencing. Samples were run on a combination of Illumina HiSeq 2500, HiSeq 4000, and NovaSeq instruments, and multiplexed using unique dual barcode indexes to avoid sample contamination and barcode hopping. Final RNA counts were generated as normalized read counts using the DEseq estimateSizeFactors function.

### Statistical and RNA-seq analysis

We evaluated key genes involved in MIF, DDT and CD74 signaling and correlated RNA levels with clinicopathologic variables using R (version 4.3.1) based packages ggplot2 (version 3.5.1) and survminer (version 0.4.9). OS was defined as the time between date of initial diagnosis and death. Deceased patients without a documented date of death were excluded. PFS was defined as the time in months between initiation of active ICI therapy and evidence of disease progression as documented in provider notes with confirmation on radiographic imaging or with biopsy. Patients who were not on active or prior ICI therapy at the time of biopsy were excluded from PFS analyses. MIF, DDT, and CD74 gene expression levels obtained from bulk RNA sequencing were used to subsequently divide patients into high and low expression groups using maximally selected log ranked statistics. Cut points were generated using the surv.cutpoint function in survminer for OS data and applied to OS and corresponding PFS data using the surv_categorize function. Kaplan Meier survival curves were generated for OS and PFS using the survfit and ggsurvplot functions, and were compared by log-rank testing. Kaplan Meier curves were also generated using median and quartile cutpoint approaches (data not shown). Tumor infiltrating immune cells were profiled using CIBERSORT, CIBERSORT abs.mode, EPIC, MCP-Counter, quanTIseq, TIMER, and Xcell methods [52, 53]. Deconvolution analysis was conducted to assess differences in immune cell type abundance between high and low CD74:MIF and CD74:DDT groups which were determined by OS-derived surv.cutpoint values.

Differential gene expression analysis was then conducted using the R based DESeq2 (version 1.40.2) package to detect differentially expressed genes (DEGs) in high CD74:MIF and high CD74:DDT cohorts referenced against their respective low cohorts [54]. Enriched gene sets were identified through Gene Set Enrichment Analysis (GSEA) using the clusterProfiler (version 4.8.2) package to evaluate expression of gene sets and pathways in the context of relevant oncologic and immune-related pathways [55–58]. GSEA of MSigDB hallmark gene sets and KEGG (Kyoto Encyclopedia of Genes and Genomes) pathways was performed [59, 60]. A shared list of DEGs between high CD74:MIF and high CD74:DDT cohorts was also generated after filtering for p-adjusted value less than 0.05 and absolute log2FoldChange values greater than 1.5 to select for significantly up-regulated and down-regulated genes and associated biological pathways. Common DEGs were subsequently visualized with Volcano Plots using the EnhancedVolcano (version 1.18.0) package.

## SUPPLEMENTARY MATERIALS


