# Supplementary material for: Prognostic and therapeutic insights into MIF, DDT, and CD74 in melanoma
Source: Oncotarget. 2024 Jul 19;15:507–20. doi: 10.18632/oncotarget.28615 (PMC11259151; doi:10.18632/oncotarget.28615)
Supplement: Supplementary file 1 [file oncotarget-15-28615-s001.pdf]

Prognostic and therapeutic insights into MIF, DDT, and CD74 in melanoma

SUPPLEMENTARY MATERIALS

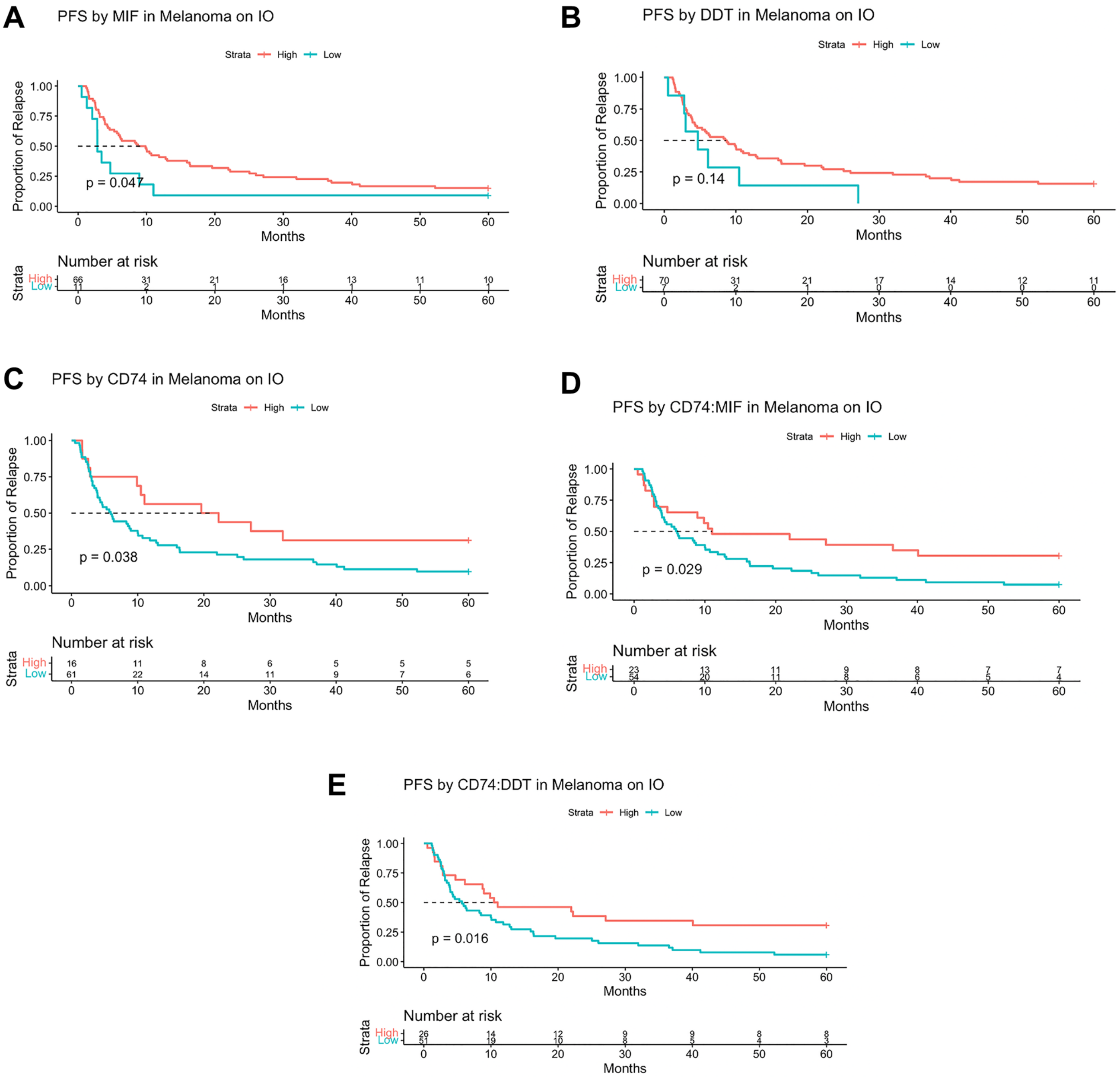

Supplementary Figure 1: PFS analyzed according to MIF, DDT, and CD74 using the PFS-derived surv_cutpoint approach in Rstudio. Elevated MIF (**A**), DDT (**B**), and CD74 (**C**) levels are associated with improved PFS (MIF $p = 0.047$, DDT $p = 0.14$, CD74 $p = 0.038$). (**D**, **E**) Elevated CD74:MIF and CD74:DDT levels are also associated with improved PFS (CD74:MIF $p = 0.029$, CD74:DDT $p = 0.016$). Number at risk refers to the quantity of eligible patients alive at each time point. IO = Immunotherapy.

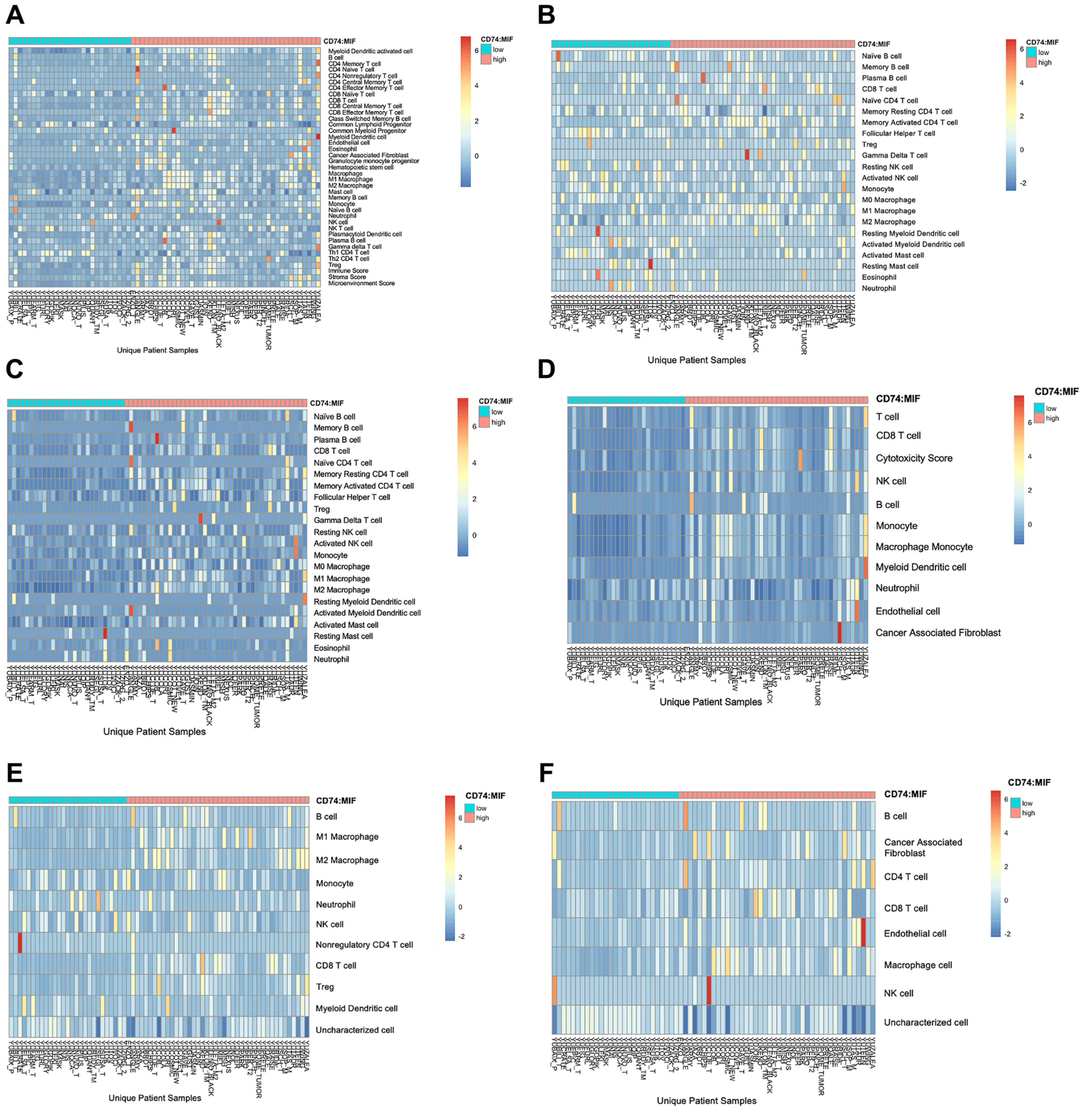

Supplementary Figure 2: An enrichment of intratumoral immune cells are present in high CD74:MIF groups when compared with low cohorts as seen with (**A**) XCELL, (**B**) CIBERSORT, (**C**) CIBERSORT abs.mode, (**D**) MCP_Counter, (**E**) quanTIseq, and (**F**) EPIC deconvolution algorithms. Cutoff for high and low values was determined using the OS-derived surv_cutpoint approach on Rstudio.

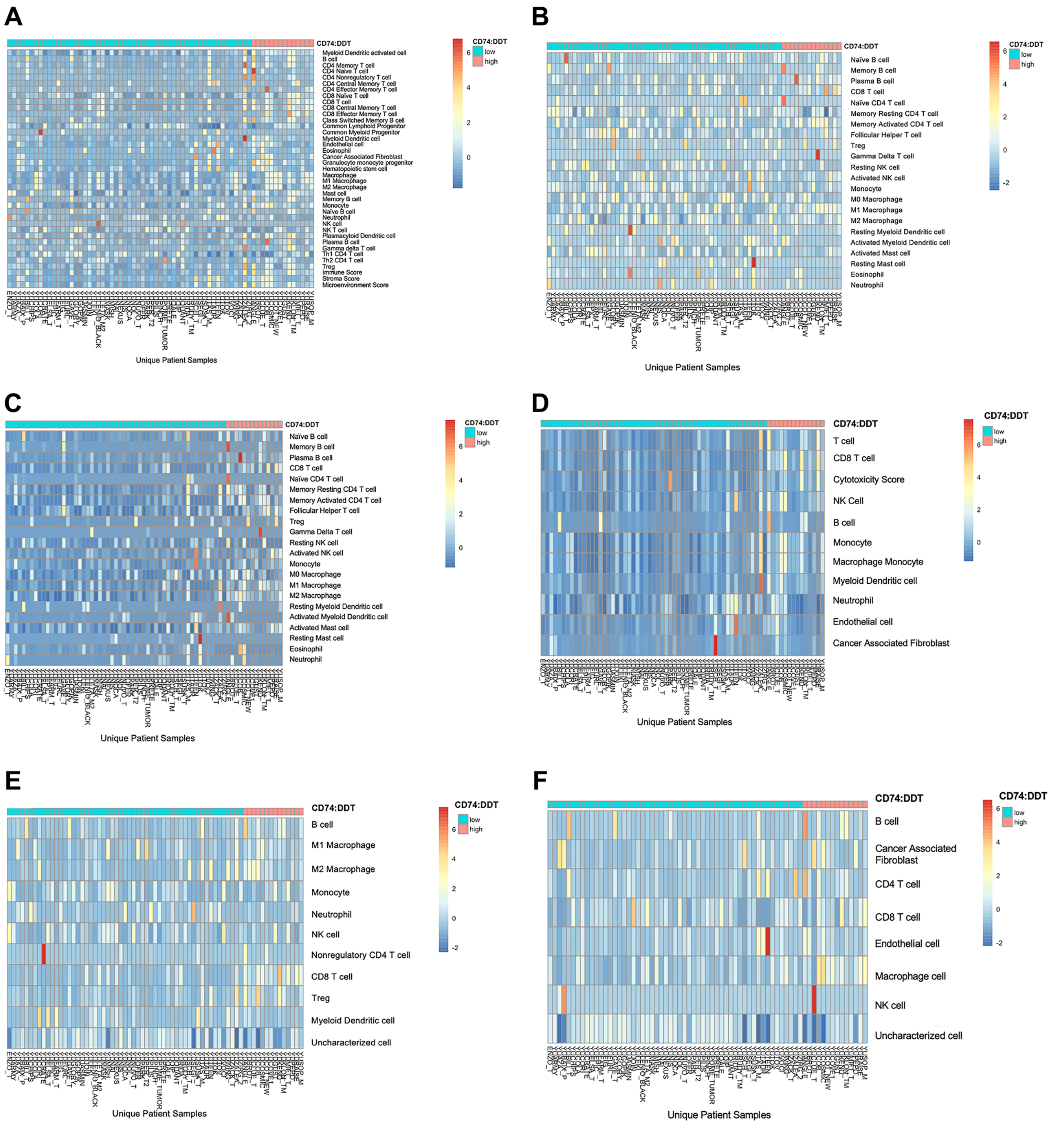

Supplementary Figure 3: An enrichment of intratumoral immune cells are present in high CD74:DDT groups when compared with low cohorts as seen with (**A**) XCELL, (**B**) CIBERSORT, (**C**) CIBERSORT abs.mode, (**D**) MCP_Counter, (**E**) quanTIseq, and (**F**) EPIC deconvolution algorithms. Cutoff for high and low values was determined using the OS-derived surv_cutpoint approach on Rstudio.

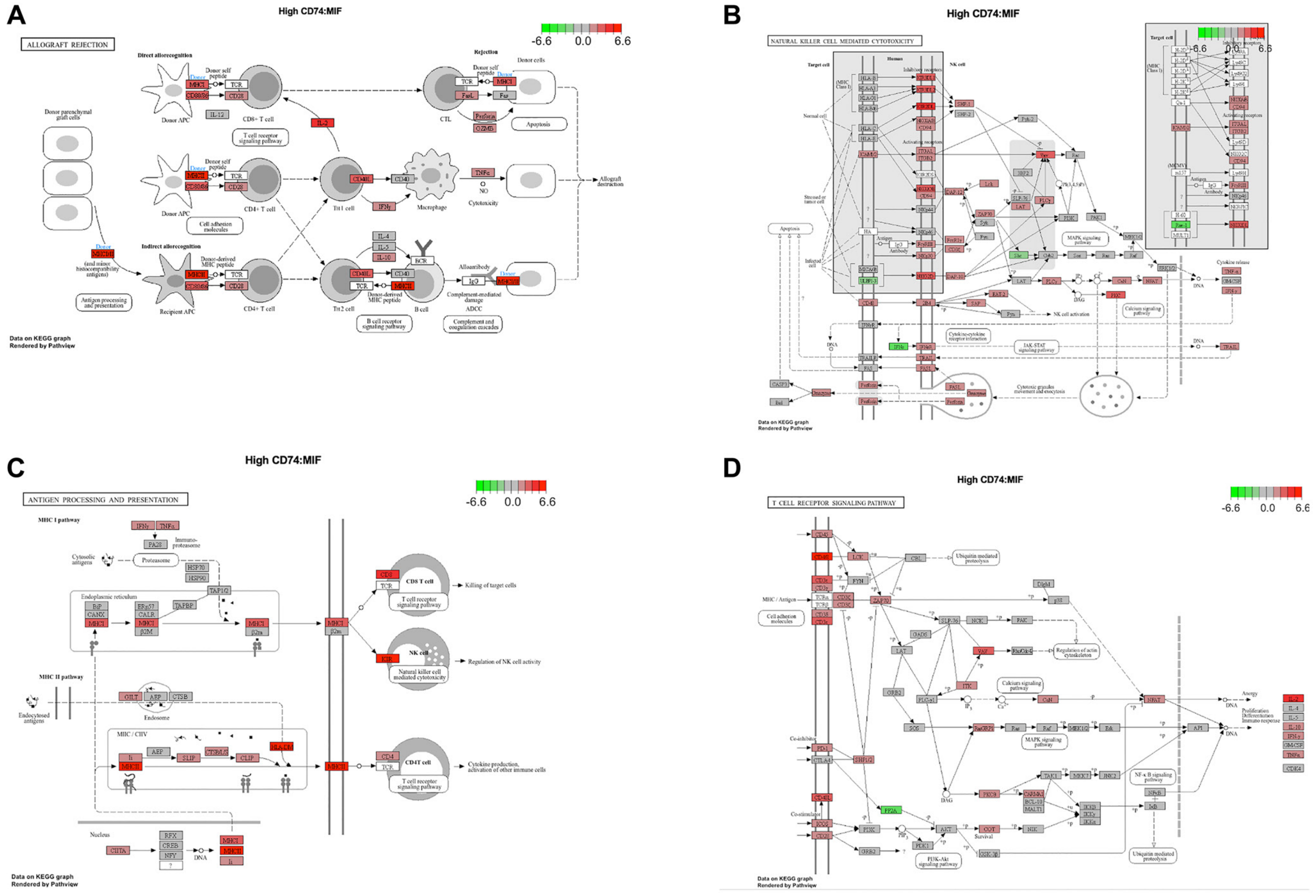

Supplementary Figure 4: Pathway analysis of differentially expressed genes in high CD74:MIF groups according to Kyoto Encyclopedia of Genes and Genomes (KEGG) Pathway Sets reveals an upregulation of immune related pathways involved in (**A**) Allograft Rejection, (**B**) Natural Killer Cell Mediated Toxicity, (**C**) Antigen Processing and Presentation, and (**D**) T cell Receptor Signaling. Upregulated genes are represented in red and downregulated genes are represented in green.

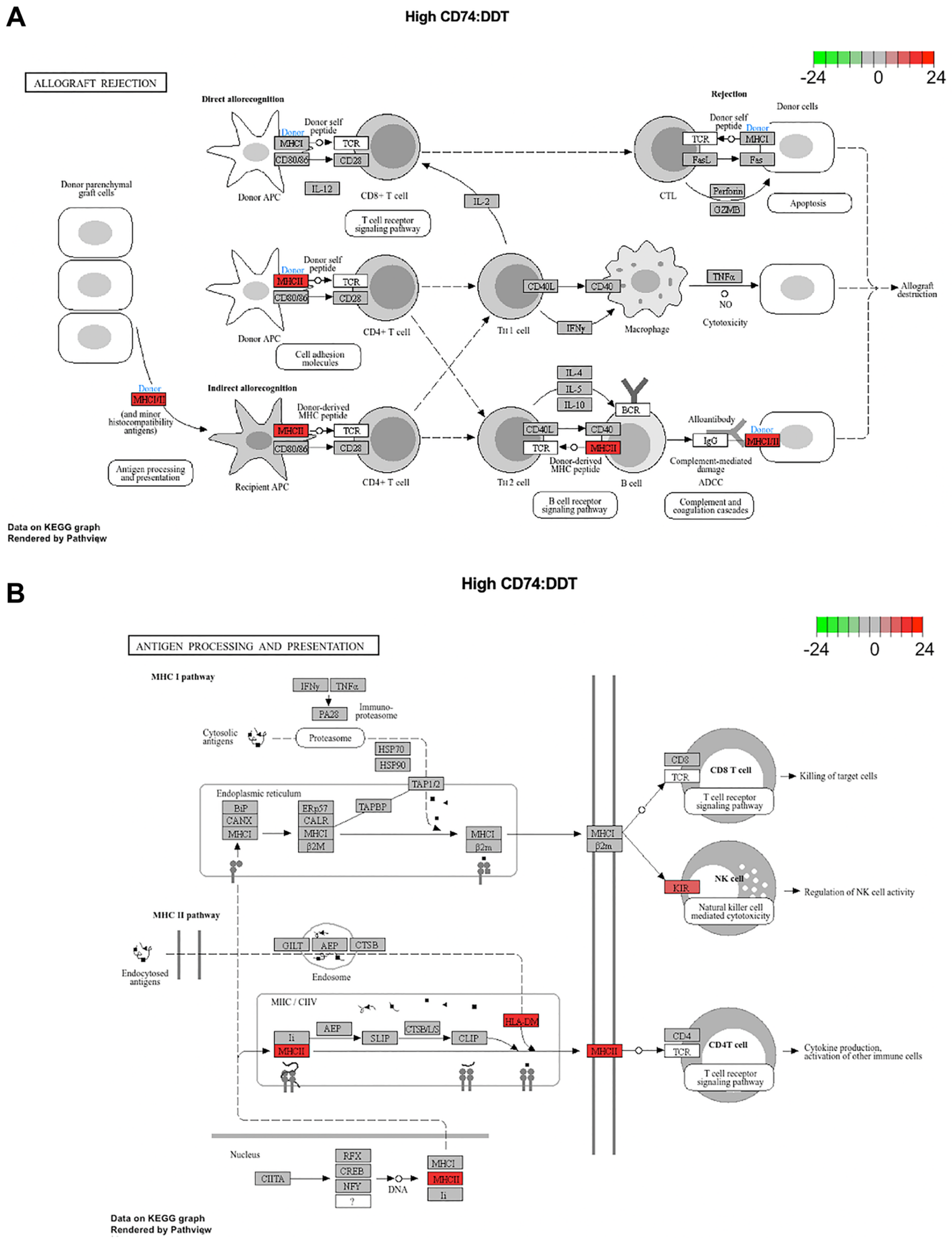

Supplementary Figure 5: Pathway analysis of differentially expressed genes in high CD74:DDT groups according to Kyoto Encyclopedia of Genes and Genome (KEGG) Pathway Sets reveals an upregulation of immune related pathways involved in (**A**) Allograft Rejection and (**B**) Antigen Processing and Presentation. Upregulated genes are represented in red and downregulated genes are represented in green.

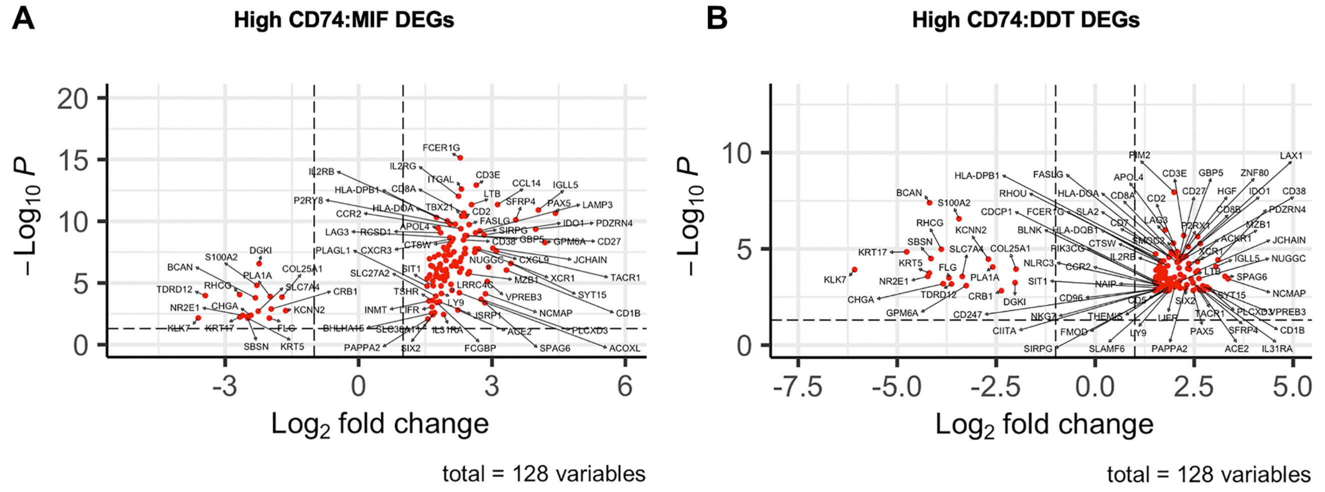

Supplementary Figure 6: Volcano Plot representations of common genes between cohorts with (**A**) high CD74:MIF and (**B**) high CD74:DDT DEGs. Prior to analysis DEGs were filtered for *p*-adjusted value < 0.05 and absolute Log2FoldChange values greater than 1.5 to select for significantly up-regulated and down-regulated biological pathways.

Supplementary Table 1: A list of shared differentially expressed genes (DEGs) between high CD74:MIF and high CD74:DDT cohorts after filtering for DEGs with a padj < −0.05 and an absolute Log2FoldChange > 1.5

	CD74_MIF_Log2FoldChange	CD74_DDT_Log2FoldChange
PIM2	1.592	1.991
APOL4	1.788	1.768
CD3E	2.645	2.235
ZNF80	2.274	2.587
LAG3	2.052	1.975
IDO1	2.822	2.653
HGF	1.769	2.348
CD2	2.405	2.067
HLA-DOA	2.173	1.926
CD8A	2.367	2.002
HLA-DPB1	1.751	1.525
CD7	1.922	1.889
P2RX1	2.129	2.183
LAX1	2.278	2.289
SMOC2	1.811	2.051
SLA2	1.957	1.863
CD8B	2.182	2.214
CD27	2.394	2.109
PDZRN4	3.981	3.100
GBP5	2.431	2.131
RHOU	1.594	1.726
FCER1G	2.286	1.656
MZB1	2.459	2.579
HLA-DQB1	1.707	1.649
FASLG	2.484	2.069
CD38	2.358	2.085
XCR1	2.622	2.363
IL2RB	1.993	1.634
CTSW	2.087	1.829
IGLL5	4.042	3.047
KCNA3	2.396	2.118
TIGIT	1.748	1.732
PTGER4	1.627	1.584
ACKR1	2.612	2.335
IL2RG	2.246	1.684
LRRC4C	2.099	2.378
RCSD1	1.843	1.535
JCHAIN	3.020	2.594
CXCR3	2.123	1.769
ACOXL	2.264	2.544
P2RY8	2.061	1.630
ITGAL	2.311	1.646
PLAGL1	1.540	1.663
LAMP3	2.727	2.148
ETV7	2.031	1.761
TBX21	2.320	1.751
SPAG6	2.838	3.278
STAT4	1.542	1.543
PTPN22	1.797	1.564
CXCL9	2.699	2.241
ZBP1	2.198	2.003
LCK	1.769	1.725
CCL5	1.661	1.528
ACAP1	1.731	1.681
NUGGC	2.923	2.621
CD3G	2.140	1.797
INMT	1.582	1.901
SLC38A11	1.706	2.421
NCMAP	2.852	3.354
TTC16	1.979	1.833
SLFN12L	1.967	1.806
C1QTNF3	2.094	1.878

JSRP1	1.818	2.135
C16orf54	1.834	1.614
TSHR	1.662	1.909
AKAP5	1.973	1.822
CXCR6	2.161	1.860
BLNK	1.602	1.504
FCGBP	1.756	2.092
LTB	2.538	2.069
CYSLTR1	1.667	1.616
CDCP1	1.822	1.611
SLC27A2	1.736	1.897
SFRP4	3.533	2.470
IKZF3	2.143	1.855
CCL14	3.125	2.102
PIK3CG	1.915	1.530
CXCL11	2.077	2.038
UBASH3A	2.311	2.022
GRIN3A	1.853	1.825
SIX2	1.672	2.314
CARMIL2	1.829	1.698
SLITRK4	2.375	1.899
CCR2	2.298	1.667
NLRC3	1.882	1.653
TRAF3IP3	1.889	1.726
SYT15	3.423	2.716
NAIP	2.064	1.664
CD5	2.307	1.872
LIFR	1.650	2.010
CD1B	3.324	2.773
VPREB3	2.892	2.822
TACR1	3.095	2.572
POU2AF1	2.491	2.146
SIT1.00	1.678	1.610
BHLHA15	1.644	2.143
SIRPG	2.631	1.881
PAPPA2	1.556	2.277
THEMIS	1.982	1.761
CD96	2.024	1.592
PLCXD3	2.756	2.886
NKG7	1.965	1.579
CD247	1.747	1.522
ACE2	2.232	2.641
LY9	1.861	1.872
FMOD	1.931	1.741
PAX5	4.426	2.474
IL31RA	1.912	2.738
CIITA	1.993	1.507
SLAMF6	2.396	1.819
CRB1	−1.966	−2.370
GPM6A	4.190	−3.260
TDRD12	−3.447	−3.631
CHGA	−2.423	−3.842
DGKI	−2.238	−2.024
FLG	−2.008	−3.694
SLC7A4	−2.259	−3.359
NR2E1	−2.607	−4.229
KRT5	−2.490	−4.193
KLK7	−3.606	−6.078
COL25A1	−1.724	−2.001
PLA1A	−1.987	−2.589
KCNN2	−1.642	−2.699
SBSN	−2.508	−4.149
KRT17	−2.670	−4.764
RHCG	−2.677	−3.889
S100A2	−2.288	−3.441
BCAN	−2.321	−4.185

There is a high concordance of similarity between upregulated and downregulated genes between both groups.
